# Identification of a Gene Sharing a Promoter and Peroxisome Proliferator-Response Elements With Acyl-CoA Oxidase Gene

**DOI:** 10.1155/PPAR/2006/71916

**Published:** 2006-12-12

**Authors:** Mst. Hasina Akter, Md. Abdur Razzaque, Liu Yang, Toshio Fumoto, Kiyoto Motojima, Tomohiro Yamaguchi, Fumiko Hirose, Takashi Osumi

**Affiliations:** ^1^Graduate School of Life Science, University of Hyogo, Hyogo 678-1297, Kamigori, Japan; ^2^International Research and Educational Institute for Integrated Medical Sciences, Tokyo Women's Medical University, Tokyo 162-8666, Shinjuku, Japan; ^3^Department of Biochemistry, Meiji Pharmaceutical University, Tokyo 204-8588, Kiyose, Japan

## Abstract

Many mammalian genes are clustered on the genomes, and hence the genes in the same cluster can be regulated through a common regulatory element. We indeed showed previously that the perilipin/PEX11*α* gene pair is transactivated tissue-selectively by PPAR*γ* and PPAR*α*, respectively, through a common binding site. In the present study, we identified a gene, named GSPA, neighboring a canonical PPAR target, acyl-CoA oxidase (AOX) gene. GSPA expression was induced by a peroxisome proliferator, Wy14,643, in the liver of wild-type mice, but not PPAR*α*-null mice. GSPA and AOX share the promoter and two peroxisome proliferator-response elements. GSPA mRNA was also found in the heart and skeletal muscle, as well as 3T3-L1 cells. GSPA encodes a protein of 161 amino acids that is enriched in 3T3-L1 cells. Even other gene pairs might be regulated through common sequence elements, and conversely it would be interesting how each gene is aptly regulated in clusters.

## INTRODUCTION

Recent analyses of human and other mammalian genomes have revealed that unexpectedly a large number of protein-coding genes are clustered [1], being arranged head-to-head, tail-to-head, or tail-to-tail. More recent comprehensive studies [2] revealed that more than 60% of the mouse genome is transcribed into RNA, often for both strands in the same regions. Many of the RNA products do not seem to code for proteins [3], and most of such noncoding RNAs are yet uncharacterized.

Given such clustered arrangements of transcribed regions, it would be inferred that two or more clustered genes (or transcriptional units) are possibly regulated by common cis-elements in considerable number of instances. Clustered genes with related functions formed by gene duplication, for example, the *β*-globin gene cluster [4] and albumin/*α*-fetoprotein gene pair [5], have been known to be regulated by common enhancers. However, it is expected that even functionally and structurally unrelated genes can be regulated by a common mechanism, simply because a regulatory element for one gene is located close to the other in a cluster. We have indeed reported that the genes of PEX11*α*, a peroxisome biogenesis factor, and perilipin, a lipid droplet-coating protein, are regulated by peroxisome proliferator-activated receptor (PPAR) subtypes through a common cis-element [6]. The PEX11*α* and perilipin genes are arranged in tandem in this order, with the same transcriptional orientation. A common peroxisome proliferator-response element (PPRE), which serves as a binding site of PPAR/RXR heterodimer [7], is located within the spacer region, 8.4 kb downstream of the PEX11*α* promoter, whereas 1.9 kb upstream of the perilipin promoter. In the liver, this PPRE confers the action of PPAR*α*, leading to the induction of PEX11*α* by the PPAR*α* ligands, peroxisome proliferators. On the other hand, in the adipose tissue, the same PPRE is recognized by PPAR*γ*, hence resulting in the expression of perilipin dependent on adipogenesis. The differential regulation of these genes is probably attained by the differential expression of the two PPAR subtypes in the liver and adipose tissue, and also by the differences in the positions and/or distances of the PPRE relative to the promoters. Differential interactions with other transcriptional factors also seem important [8]. One of such factors is NF-I, which is required for the activation of perilipin gene by PPAR*γ*, but not for that of PEX11*α* gene by PPAR*α*.

To examine the generality of such common regulatory mechanisms for clustered genes, we searched in the mouse EST databases for transcripts that start from positions close to the promoters of known PPAR target genes. We report here the identification of a gene that is transcribed in the opposite orientation from the promoter of acyl-CoA oxidase (AOX) gene, a canonical target of PPAR*α*. This gene also shares the PPAR*α* target sites with the AOX gene.

## MATERIALS AND METHODS

### Construction of reporter plasmids

**Figure 1:**
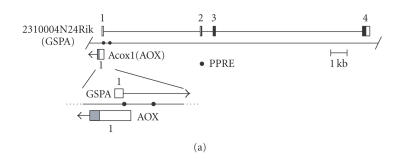

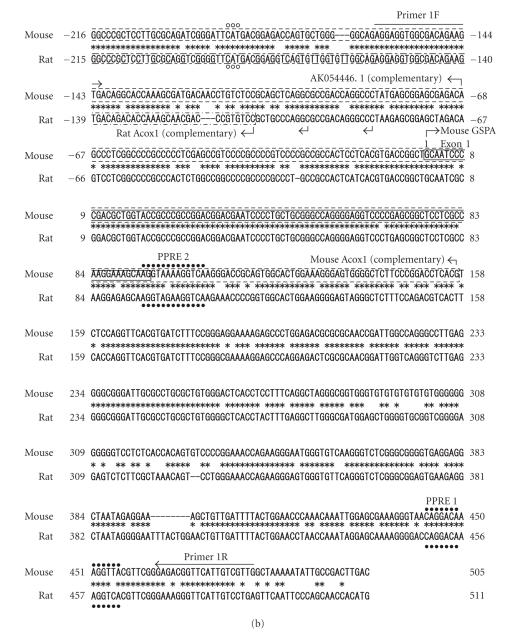

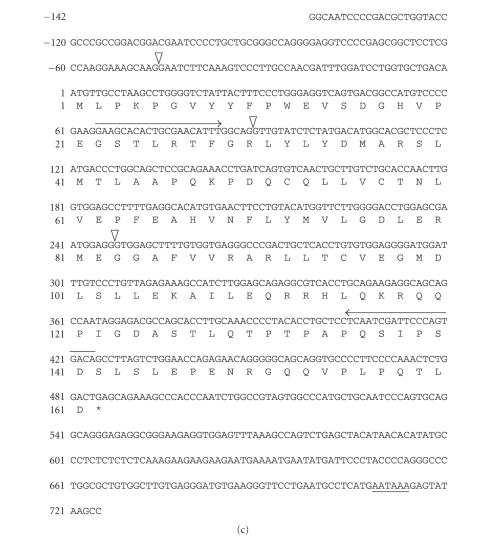
Identification of GSPA in the mouse genome. (a) GSPA is positioned closely adjacent to the AOX gene in the opposite orientation. Exons are shown with boxes and numbered. For AOX gene, only exon 1 is presented, starting from the minor transcriptional initiation site. Open, filled, and gray areas indicate noncoding, GSPA-coding, and AOX-coding regions, respectively. Closed circles denote two PPRE-like motifs. In the enlarged view, exon 1 of each gene is depicted together with the transcriptional orientation shown with horizontal arrow. (b) Alignment of mouse and rat genomic sequences around the transcriptional initiation sites of GSPA and AOX genes including the two PPRE-like motifs. Nucleotide numbers are shown starting from the transcriptional initiation site of GSPA, with increasing numbers in the direction of GSPA transcription. Exon 1 of GSPA, mouse AOX, and rat AOX are boxed with solid, broken, and chain lines, respectively. Major and minor transcriptional initiation sites of the genes are indicated with large and small arrows, respectively, pointing the direction of transcription. Closed circles, asterisks, and dashes denote two PPRE-like motifs, nucleotides conserved between mouse and rat, and gaps, respectively. Horizontal arrows indicate sites of PCR primers 1F and 1R, used for reporter construction. (c) cDNA and predicted amino acid sequences of mouse GSPA. Nucleotides and amino acids are numbered taking the first letter of the predicted initiation codon and initiator methionine as 1, respectively. Nucleotides of the 5′ noncoding region are indicated with negative numbers. Amino acids are presented with single-letter codes, asterisk indicating a stop codon. Triangle denotes the position of intron insertion. Horizontal arrows and underline indicate the sites of primers for RT-PCR and polyadenylylation signal, respectively.

We first searched for a gene that is mapped close to established PPAR target genes in the mouse genome. Using NCBI Mouse Genome Resources, we found a gene, named GSPA in this work, which is located just upstream of the AOX gene in the opposite orientation (see “results”). For constructing reporter plasmids, we amplified appropriate DNA fragments by PCR from a mouse BAC clone, RP23-174D24. For GSPA, fragment encompassing positions −161 through 483 (for position numbers, see Figure 1(b)), which contained the basal promoter, exon 1, and the early part of intron 1, was amplified with primers 1F, 5′-AGGAGGTGGCGACAGAAGTG-3′ and 1R, 5′-CAACGACAATGAACCGTCTCC-3′. This fragment was inserted into the EcoRV site of pBluescript KS(-) (Stratagene), yielding plasmids, pBSfr1-1 and pBSfr1-2. The fragment was inserted in opposite orientations in these plasmids, the HindIII site of the multicloning region being on the upstream and downstream sides relative to the insert, respectively. Another genomic fragment, encompassing from the position 340 bp upstream of intron 1/exon 2 boundary to the 39th position of exon 2 of GSPA, was amplified using a primer pair 2F, 5′-ACCTCTGCAGGCCCATGCTG-3′ and 2R, 5′-ACCAGGATCCAAATCGTTGGC-3′. This fragment was inserted into the EcoRV site of pBluescript-KS(-), yielding pBSfr2, in which the HindIII site of the vector is located on the upstream side. The insert of pBSfr1-2 was cleaved out with HindIII and SalI, and inserted between the HindIII and SalI sites of pBSfr2. The resulting plasmid, pBSfr1/2, contains GSPA sequences for the basal promoter, exon 1, parts of intron 1, one of which containing the two putative PPREs, and a part of exon 2 before the putative initiation codon. A stretch of 5133 bp in the middle portion of intron 1 was removed, to reduce the plasmid size. It was expected that transactivation by PPAR*α* would be observed even with this partially deleted construct, if the putative PPREs have sufficient functions as in the rat AOX gene. pBSfr1/2 was cleaved with ApaI, blunt-ended with Klenow fragment, and then cleaved with BamHI. The GSPA-derived sequence was isolated and inserted between the SmaI and BglII sites of a promoter-less luciferase reporter vector, pGVBΔ, in which the SV40 small T intron was eliminated from pGVB (Toyo Ink) to prevent aberrant splicing [9]. The final product, pGSPAluc, was used for the reporter assay to monitor the activation by PPAR*α*. For constructing the AOX reporter, the insert of pBSfr1-1 was cleaved out with SmaI and HindIII, and inserted between the SmaI and HindIII sites of pGVBΔ, yielding pMmAOXluc, where the mark “Mm” was attached for discriminating the construct from the rat AOX reporters already described [10]. In this construct, the genome-derived sequence was inserted in the vector, in the same orientation as that of AOX, that is, reverse to that of GSPA. A truncated reporter plasmid, pMmAOXBluc, was constructed by removing the region between positions 21 and 483 from pMmAOXluc, exploiting an internal KpnI site (see Figure 1(b)). pMmAOXBluc lacked both putative PPREs, while retaining the sequence corresponding to the rat AOX minimal promoter [11].

### Site-directed mutagenesis

Mutant reporter constructs in which the putative PPREs, PPRE-1, PPRE-2, or both, were destroyed were created by the PCR-based overlap extension method [12]. The following oligonucleotides carrying base substitutions in the PPRE-1 or PPRE-2 portion were used: mutPPRE-1F, 5′-AAAGGGTAACctcgagAAGGTTACGT-3′; mutPPRE-1R, ACGTAACCTTctcgagGTTACCCTTT-3′; mutPPRE-2F, 5′-AAAGCAAGGTAAAAGcgatAGGGAC-3′; and mutPPRE-2R, 5′-GTCCCTatcgCTTTTACCTTGCTTT-3′, where underlines denote the sequences corresponding to PPREs, small letters representing mutated bases. Two other primers designed so as to match the vector sequences outside the inserts were also used: pGVB-uni, 5′-TGTATCTTATGGTACTGTAACTG-3′, positioned upstream of the polylinker region; Luc-rev, 5′-ATGTTTTTGGCGTCTTCCA-3′, positioned just downstream of the luciferase initiation codon in the antisense direction. For PPRE-1 mutation in the GSPA reporter, the first PCR was performed using primer pairs, pGVB-uni/mutPPRE-1R and mutPPRE-1F/Luc-rev, employing pGSPAluc as a template. The PCR products were mixed, denatured, reannealed, and subjected to the second round of PCR, using a primer pair, pGVB-uni/Luc-rev. The product was digested with PstI, and used to replace the corresponding PstI/PstI region of pGSPAluc, yielding the PPRE-1 mutant construct, pGSPA(mutPPRE-1)luc. For PPRE-1 mutation in the AOX reporter, oligonucleotide pairs pGVB-uni/mutPPRE-1F and mutPPRE-1R/Luc-rev were used as primers, and pMmAOXluc as a template, in the first round of PCR. The second PCR was performed as above, and the product was used to replace the SmaI/HindIII region of pMmAOXluc, yielding pMmAOX(mutPPRE-1)luc. The PPRE-2 mutants, pGSPA(mutPPRE-2)luc and pMmAOX-(mutPPRE-2)luc, were constructed by similar procedures as above, except for using mutPPRE-2F and mutPPRE-2R primers instead of mutPPRE-1F and mutPPRE-1R, respectively. The double mutation constructs, pGSPA(mutPPRE1/2)luc and pMmAOX(mutPPRE1/2)luc, were created by mutating PPRE-2 of pGSPA(mutPPRE-1)luc and pMmAOXluc-(mutPPRE-1)luc. All PCR procedures were performed using KOD-plus DNA polymerase (Toyobo), and presence of desired mutations and absence of unexpected mutations were confirmed by DNA sequencing.

### Reporter assays

HeLa cells were cultured in 12-well plates and transfected with DNA by a calcium phosphate method. For each well, transfection was performed using plasmid mixtures composed of 0.8 *μ*g of a reporter plasmid, 0.1 *μ*g of a PPAR*α* expression vector, pNCMVPPAR*α*, as necessary, and 0.2 *μ*g of pCMV*β* as an internal control. Total amount of DNA was kept at 1.5 *μ*g/well by the addition of appropriate amount of an empty vector, pCMX. Other experimental conditions were as described previously [6].

### Reverse transcription (RT)-PCR

Expression of GSPA in the tissues of wild-type and PPAR*α*-null mice was estimated by RT-PCR. Mice were fed *ad libitum* with a laboratory chow containing or not containing a peroxisome proliferator, Wy14, 643. RNA from undifferentiated and differentiated 3T3-L1 cells were also analyzed. The following primers were used: GSPA-F, 5′-GAAGCACACTGCGAACATTTG-3′; and GSPA-R, 5′-TGTCACTGGGAATCGATTGAG-3′. Other experimental conditions and primer sequences were as described previously [13].

### Western blotting

Expression of GSPA protein (GSPAp) in mouse tissues as well as 3T3-L1 preadipocytes and adipocytes were estimated by Western blotting. An antibody to GSPAp was raised in rabbits, using glutathione S-transferase (GST)-fused GSPAp expressed in *Escherichia coli*. Proteins were separated by SDS-PAGE using a 13% polyacrylamide gel. Other experimental procedures were as described previously [13].

### Electrophoretic gel-mobility shift assay (EMSA)

A ^32^P-labeled double-stranded oligonucleotide containing the rat AOX PPRE-1 was used as a probe, as described in [10]. Oligonucleotides encompassing mouse AOX/GSPA PPRE-1, PPRE-2, and their mutant versions were used as competitors. They were composed of the following sequences and the respective complements: PPRE-1, 5′-AAAGGGTAACAGGACAAAGGTTACGT-3′; mutPPRE-1, 5′ -AAAGGGTAACctcgagAAGGTTACGT-3′; PPRE-2, 5′- AAAGCAAGGTAAAAGGTCAAGGGAC-3′; and mutPPRE-2, 5′-AAAGCAAGGTAAAAGcgatAGGGAC-3′. Assays were carried out as described in [10], using a maltose binding protein (MBP)-fused PPAR*α* and GST-fused RXR*α*.

## RESULTS

### Identification in silico of a gene sharing the promoter with the AOX gene

We searched for a gene that is located near a known PPAR target gene in the NCBI mouse genome map. Several EST clones derived from the same transcript (eg, AK009156 (Riken 2310004N24)) were found to be mapped in the upstream region of AOX gene (Acox1; GI:66793428) (Figure 1(a)), on the mouse chromosome 11. The gene corresponding to Riken 2310004N24 and the AOX gene seem to share the promoter, being transcribed in the opposite orientations. Hence, we named the gene corresponding to Riken 2310004N24, GSPA (a gene sharing a promoter with the AOX gene). GSPA is constituted by four exons, spanning 17, 268 bp. Upon closer inspection of the promoter region, the first exons of GSPA and AOX overlap to each other (Figure 1(b)), according to the RefSeq of AOX mRNA (NM 015729.2). However, a vast majority of mouse AOX EST clones start at more downstream positions (eg, AK054446.1; Figure 1(b)), and hence, with regard to the major AOX start site, the GSPA and AOX genes are arranged head-to-head, separated by a small space. The nucleotide sequences of the GSPA/AOX promoter regions are well conserved between mouse and rat (chromosome 10). The major transcription start site of the rat AOX gene has been mapped more downstream as compared with that of the mouse AOX gene, with several minor initiation sites positioned more upstream [14], close to the major start sites of mouse AOX gene. GSPA has an ORF starting from the first ATG triplet located in exon 2, encoding a hypothetical protein of 161 amino acid residues (BAB26112; Figure 1(c)), which is the longest ORF predictable from the cDNA sequence.

### GSPA is a target gene of PPAR*α*

The PPRE of rat AOX gene is best characterized among others, being located 560 to 572 nucleotides upstream of the major cap site, corresponding to PPRE-1 in Figure 1(b) [11, 15]. This element is conserved in the mouse genome, with a single nucleotide deviation. These elements of rat and mouse have one and two mismatches as compared with the consensus PPRE sequence (AGGTCA N AGGTCA) [7], respectively. In the mouse genome, another PPRE-like sequence, PPRE-2, was found at positions 161 to 173 nucleotides upstream of the AK054446.1 start site. This element was conserved in the rat with a single base mismatch, and the deviation from the PPRE consensus sequence is one for the mouse and two for the rat, respectively. While PPRE-1 is located in the first intron with respect to GSPA, PPRE-2 encompasses the exon 1/intron 1 junction of GSPA. Previous studies established that the rat AOX gene is regulated by PPAR*α* through the PPRE-1 [10, 11, 15], while the role of PPRE-2 was not noted. Because the mouse AOX gene is also regulated by PPAR*α*, PPRE-1 and/or PPRE-2 were likely to serve as functional PPREs, and it was further expected that GSPA is also regulated by the same mechanism through the same PPRE(s).

**Figure 2:**
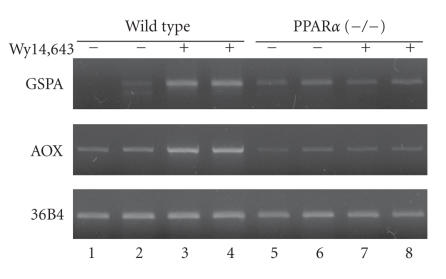
GSPA is a target gene of PPAR*α*. RT-PCR was performed with RNA samples from two animals for wild type or PPAR*α*-null mice treated or not with Wy14,643. A ribosomal subunit gene, 36B4, was used as a control unaffected by Wy14, 643. PCR was performed for 30, 22, and 26 cycles for GSPA, AOX, and 36B4, respectively. Other experimental conditions were as described in [13].

To examine this possibility, we studied the induction of GSPA expression by a peroxisome proliferator, Wy14,643, in comparison with that of AOX. Liver RNA was prepared from the wild-type and PPAR*α*-knockout mice, fed with or without Wy14,643, and analyzed for gene expression by RT-PCR (Figure 2). In the wild-type mice, GSPA was markedly induced by the drug, whereas in the PPAR*α*-null mice, no induction was observed. For AOX, PPAR*α*-dependent induction by Wy14,643 was confirmed as reported previously [6, 16]. Thus, GSPA is a bona fide target of PPAR*α*.

### Both PPRE-1 and PPRE-2 are involved in the transcriptional regulation of GSPA and AOX

**Figure 3:**
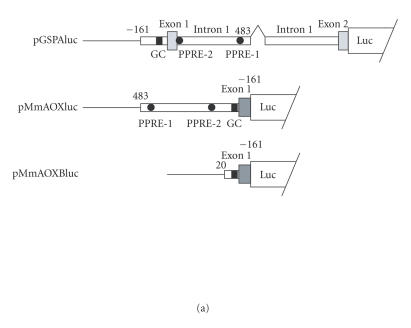

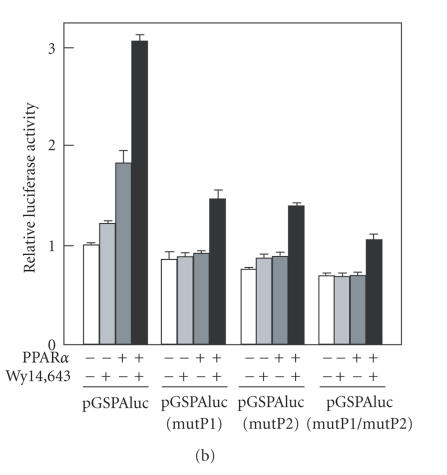

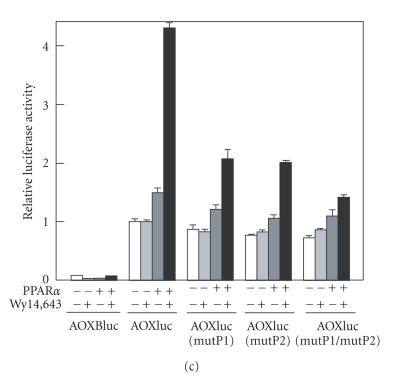
Both PPREs contribute to the PPAR*α*-dependent transactivation of GSPA and AOX. (a) Schematic view of the reporter constructs. Upstream and intron regions are depicted with horizontally long boxes, whereas exons with vertically long gray boxes. Nucleotide numbers correspond to those in Figure 1(b). The −161/483 region was included in common in both pGSPAluc and pMmAOXluc in opposite orientations. Only parts of 5′ noncoding stretches of exon 2 of GSPA and exon 1 of AOX were included in the respective reporters. In pGSPAluc, whole exon 1 that is noncoding and intron 1 were included, while a 5.1 kb region in the middle of intron 1 was omitted. pMmAOXBluc was also prepared as to lack both PPRE-1 and PPRE-2, but retained the GC-rich region. (b) and (c) Reporter assays for GSPA and AOX expressions, respectively. pGSPAluc, pMmAOXluc, and their mutants involving PPRE-1 (mutP1), PPRE-2 (mutP2), or both (mutP1/mutP2) were transfected into HeLa cells with or without a PPAR*α* expression plasmid, and after transfection, the cells were cultured in the presence or absence of Wy14, 643. For AOX, a minimal promoter vector, pMmAOXBluc, was also employed. Letters “pMm” are omitted from the names of plasmids in the figure. In both (b) and (c), the luciferase activities are shown as relative values, taking the values of respective wild-type constructs in the absence of PPAR*α* expression plasmid and Wy14643, as 1. Mean values of three independent assays are given, together with standard deviations. The actual mean luciferase activity values in the presence of both PPAR*α* and Wy14, 643 were 2.15×10^6^ and 2.37×10^7^ luciferase units for pGSPAluc and pMmAOXluc, respectively.

To assess the roles of these putative PPREs, we performed gene reporter assays with respect to both AOX and GSPA transcriptional orientations. For AOX, an upstream region containing both PPRE-1 and PPRE-2, as well as the basal promoter region (nucleotide positions 483 to −161 in Figure 1(b)) was placed upstream of the luciferase reporter gene (Figure 3(a)). On the other hand, for GSPA, a region encompassing the basal promoter starting at −161, exon1, intron 1, and the early part of exon 2 up to 8 nucleotides before the initiation codon was inserted in a luciferase reporter vector. The inserts were oriented so that transcription would occur in the same directions as those of natural AOX and GSPA, respectively. Mutants were created in these reporter plasmids, in which one of the half-sites was broken for PPRE-1, PPRE-2, or both. For AOX, a reporter construct carrying only the minimal promoter (positions 21 to −161 in Figure 2), pMmAOXBluc, was also created. In reporter assays with HeLa cells, the reporter expression was significantly enhanced by cotransfection of a PPAR*α* expression vector, which was further promoted by the addition of Wy14,643 for both AOX and GSPA (Figures 3(b) and 3(c)). The activation by PPAR*α* was significantly reduced by a single mutation of either PPRE-1 or PPRE-2, and further diminished by the double mutations involving both PPRE-1 and PPRE-2, for both AOX and GSPA. The residual transactivation by PPAR*α* and Wy14,643 of the double mutant construct was possibly due to yet uncharacterized element(s) in the genome region studied, or cryptic PPRE(s) in the vector. These results suggest that PPRE-1 and PPRE-2 function in the transcriptional activation by PPAR*α* for both AOX and GSPA, synergistically. It should be noted that the luciferase activity of pMmAOXluc was about 10 times higher than that of pGSPAluc, for the values in the presence of both PPAR*α* and ligand. Thus, the shared promoter functions much more efficiently for transcription in the direction of AOX than that of GSPA. This result was consistent with that of RT-PCR (Figure 2), in which GSPA required more cycles of PCR as compared with those for AOX, to obtain comparable intensities of signals.

### PPAR*α*/RXR*α* heterodimer binds to both PPRE-1 and PPRE-2

**Figure 4:**
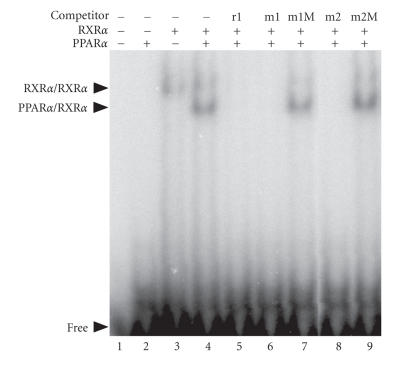
Both PPRE-1 and PPRE-2 serve as effective binding sites for the PPAR*α*/RXR*α* heterodimer. EMSA was performed using MBP-PPAR*α* and GST-RXR*α* expressed in *E coli*, using the rat AOX PPRE (corresponding to PPRE-1), as a probe. Competitors were rat AOX PPRE (unlabeled probe; r1), mouse PPRE-1 (m1) or its mutant (m1M), and mouse PPRE-2 (m2) or its mutant (m2M). Shifted bands with PPAR*α*/RXR*α* heterodimer and RXR*α* homodimer are indicated, together with that of free probe.

To examine whether these putative PPREs are recognized by PPAR*α*/RXR*α* heterodimer, we performed EMSA, using fusion proteins MBP-PPAR*α* and GST-RXR*α*. Rat AOX PPRE was used as a probe, and the wild type as well as mutant PPRE-1 and PPRE-2 were tested for the ability to compete with the probe for binding. Under the experimental conditions, PPAR*α* alone did not exhibit a band with the probe, though RXR*α* did, probably representing homodimeric binding (Figure 4, lane 3). Mixed addition of PPAR*α* and RXR*α* yielded another band corresponding to the heterodimer (lane 4). This band was efficiently competed by the unlabeled probe itself, PPRE-1, and PPRE-2 (lanes 5, 6, and 8), but not by the mutant sequence of PPRE-1 or PPRE-2 (lanes 7 and 9). Thus, both PPRE-1 and PPRE-2 served as efficient PPAR*α*/RXR*α* binding sites in vitro.

### Expression of GSPA transcript and the protein product

**Figure 5:**
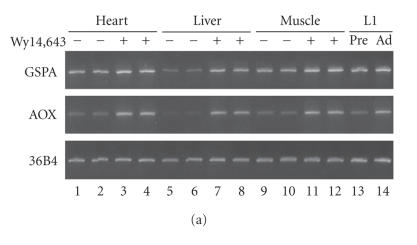

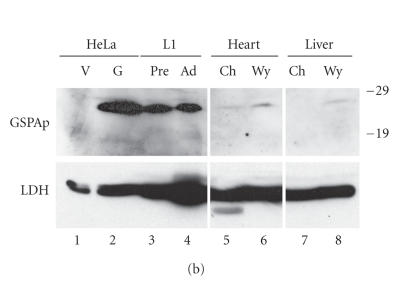
GSPA mRNA is expressed in a wide range of PPAR-expressing tissues and cells, while GSPA protein is exceedingly abundant in 3T3-L1 preadipocytes and adipocytes. (a) RT-PCR of RNA samples from the mouse heart, liver, and skeletal muscle, as well as 3T3-L1 preadipocytes (Pre) and adipocytes (Ad). Wild-type mice were fed with or without Wy14, 643, two animals being used for each condition. RT-PCR was performed as in Figure 2. (b) Western blotting of protein samples from the mouse heart and liver, as well as 3T3-L1 preadipocytes (Pre) and adipocytes (Ad). Extracts prepared from HeLa cells transfected with a GSPA-expression vector (G) and an empty vector (V) were analyzed in parallel to reveal the band position of GSPAp. Ch and Wy, tissue extracts from the mice fed normal chow or Wy14, 643-containing diet, respectively. Anti-GSPAp antiserum was used at 200-fold dilution. Membrane was reprobed with anti-lactate dehydrogenase (LDH) antibody as a loading control. Other conditions were as described previously [13]. All samples were analyzed in a single gel. Positions of size markers (in kd) are shown on the right.

PPAR*α* is also abundantly expressed in mouse tissues other than the liver, for example, the heart [15]. In addition, adipose tissue is a major site of PPAR*γ* action. Accordingly, we examined the expression of GSPA in the mouse heart and skeletal muscle, as well as 3T3-L1 preadipocytes and adipocytes, in comparison with AOX expression (Figure 5(a)). By RT-PCR, the AOX transcript was found to be induced by Wy14, 643 in the heart and skeletal muscle as in the liver, being consistent with a previous result [13]. In 3T3-L1, AOX RNA was significantly increased upon differentiation. The GSPA transcript was also found in all these tissues and cells, at comparable levels as that in the liver. Fold induction of GSPA RNA by Wy14,643 in the heart and skeletal muscle was smaller than that in the liver, due to higher basal expression in the heart and skeletal muscle. Similar levels of GSPA mRNA were detected for differentiated and undifferentiated 3T3-L1 cells. Thus, the basal expression of AOX and GSPA seems to be differentially regulated in different tissues and cells, albeit directed by the common promoter.

We next examined whether GSPA encodes a protein. For this purpose, we raised an antibody to predicted GSPA protein product (GSPAp), using a GST-GSPAp fusion protein expressed in *E. coli* as an antigen. The antiserum recognized GSPAp effectively, judged by the reactivity with both the fusion proteins of GSPAp with GST and GFP (data not shown). Presence of GSPAp was examined by Western blotting, for protein samples from the heart and liver of mouse fed with or without Wy14, 643, as well as 3T3-L1 preadipocytes and adipocytes. The antibody recognized an extra band for the extract of HeLa cells transfected with a GSPA expression vector as compared with that of the control cells (Figure 5(b); lanes 1 and 2). We judged this band to be representing GSPAp, although the estimated size (27 kDa) of the band was apparently larger than the calculated molecular mass of GSPAp (18.1 kDa). A band was detected for 3T3-L1 samples at the same position with that of GSPAp, at similar levels in the adipocytes and preadipocytes, consistent with the result of RT-PCR. A much fainter band of the same size was also observed for the heart and liver samples, apparently being induced by Wy14, 643. On the other hand, a corresponding band was not detected for skeletal muscle (data not shown). Thus, GSPA indeed encodes a protein product, but the content of the protein is highly variable among cell types, despite comparable mRNA levels.

## DISCUSSION

In the present paper, we have identified a mouse gene, GSPA, as a novel target of PPAR*α* on the mouse genome. GSPA is located closely adjacent to the AOX gene, transcribed in the orientation opposite to the latter. The transcriptional start site of GSPA is separated by less than 70 nucleotides from the predominant start site of AOX, or the transcripts of these genes even overlap, with regard to the minor AOX transcript. Hence, these genes are driven by a common promoter, which is GC-rich, while lacking a TATA-box. It has been pointed out that such TATA-less promoters often confer bidirectional transcription from less defined initiation sites [17, 18].

GSPA and AOX also share the PPREs. Two PPREs, PPRE-1 and PPRE-2, were found in the first intron of GSPA, whereas in the upstream region of AOX major transcriptional start site. Both of them are functional, acting synergistically in driving transcription of both GSPA and AOX. In the previous studies on the rat AOX, only an element corresponding to PPRE-1 was noted in gene reporter assays [11]. As compared with the idealized sequence of nuclear receptor-binding half-site, AGGTCA, mouse PPRE-1 is deviated at two positions, one in each half-site, whereas mouse PPRE-2 at only one position. On the other hand, rat PPRE-1 deviates by only one nucleotide from the consensus, while rat PPRE-2 carries two mismatches in one of the half-sites. These differences in the nucleotide sequences probably result in the different functions of these elements in the transcriptional regulation in the two species.

This is the second example of PPRE(s) shared by two genes. In the first case, the PEX11*α*/perilipin gene pair, the two genes are oriented in the same direction, and each gene is activated by PPAR*α* and PPAR*γ*, selectively in a tissue-specific manner [6]. In contrast, in the present case, the two genes are oriented in the opposite directions, and both genes are activated by PPAR*α*. In view of the recent reports that many mammalian genes are clustered in the genomes [1], and more than 60% regions of the mouse genome are transcribed [2], any PPREs as well as other transcriptional regulatory sites can be by chance positioned close to more than two genes. Hence, even more cases of shared regulatory elements would be found in future. Indeed, for example, a bidirectional promoter has been recently reported for Gabp*α*/ATP synthase coupling factor 6 genes [19]. If the neighboring genes are functionally related, they would be adequately regulated by similar mechanisms through common regulatory elements. Neighboring genes, however, might not necessarily have related functions. In such situations, a pair of genes must be regulated independently, and hence the influence of a given regulatory element must be restricted for one gene, whereas the other gene must appropriately be insulated from it. Elements having functions similar to those of “insulators” or “enhancer blockers,” which are usually imagined to function in blocking long-range enhancer actions [20], might also be involved in more nearby regulatory interactions.

Despite the sequence conservation in the GSPA/AOX promoter region between mouse and rat, it is not clear at present whether GSPA is transcribed into RNA in the rat. Although considerable number of cDNA sequences apparently derived from this genomic region have been deposited in the rat EST databases, most of them are annotated to be oriented opposite to the mouse GSPA. In human, on the other hand, an EST clone homologous to GSPA is found in the database (BC047782). In addition, another EST sequence (AK097104) has been deposited, which further extends than the mouse GSPA cDNAs on the 3′ side, continuing into the exon sequences of neighboring gene, CDK3. It seems doubtful whether the latter human cDNA represents a physiologically relevant transcript. Sequence of the promoter region of human AOX is less conserved as compared with the mouse and rat counterparts, neither PPRE-1 nor PPRE-2 being retained. Human AOX is not likely to be induced by peroxisome proliferators [21], though a potential PPRE sequence has been noted in a far upstream region [22].

We found that GSPA is widely expressed in tissues where PPAR*α* plays a regulatory role, such as liver, heart, and skeletal muscle. The dependence on PPAR*α* ligand was less significant in the heart and skeletal muscle than in the liver, due to higher basal expression. GSPA is also expressed in 3T3-L1 cells, though apparently independent of differentiation. Hence, it is not clear whether PPAR*γ* is involved in the regulation of GSPA expression in these cells. Thus, GSPA is possibly expressed in even other tissues, though the expression may not be activated by PPARs. On the other hand, we found significant expression of the GSPA protein product in 3T3-L1 adipocytes and preadipocytes. In the heart and liver, the protein abundance was much lower, despite the comparable mRNA levels. Thus, GSPA expression must also be regulated at a posttranscriptional level.

What would be the function of GSPA? In the GSPAp amino acid sequence, neither a known protein motif nor a predictable membrane-spanning domain was noted. A GFP-fused version of GSPAp expressed in HeLa cells were distributed throughout the cells, without accumulating in any subcellular compartments (data not shown), suggesting a cytosolic nature of the protein. Enrichment of GSPAp in 3T3-L1 cells over other tissues seems promising for the functional studies. It would be an interesting issue whether the abundant expression of GSPAp is characteristic in the adipocyte lineage. Other important questions would be how GSPAp expression is posttranscriptionally regulated, and whether the protein is accumulated in the liver, heart, and other tissues under specific conditions. It should be noted that, for the homologous human cDNAs, an even shorter protein sequence of 122 amino acid residues, 62% identical with the mouse sequence, is predicted, due to an in-frame stop codon at a more upstream position. Thus, it is questionable whether the function of GSPA is conserved in human. The function of GSPA should carefully be investigated also from an evolutionary point of view.
